# Inhibition of microRNA-15 protects H9c2 cells against CVB3-induced myocardial injury by targeting NLRX1 to regulate the NLRP3 inflammasome

**DOI:** 10.1186/s11658-020-00203-2

**Published:** 2020-02-19

**Authors:** Ru Tong, Tiewen Jia, Ruijie Shi, Futang Yan

**Affiliations:** 1 Laboratory Dept., Second Hospital of Shanxi Medical University, Taiyuan, 030001 Shanxi China; 2 Laboratory Dept., Shaanxi Provincial People’s Hospital, No. 256, West Youyi Road, Xi’an, 710068 Shaanxi province China

**Keywords:** Coxsackievirus B3, Viral myocarditis, MiR-15, NLRX1, NLRP3 inflammasome

## Abstract

**Background:**

Viral myocarditis (VMC) is a type of cardiac inflammation that is generally caused by coxsackievirus B3 (CVB3) infection. Several MicroRNAs (miRNAs) are known to play crucial roles in VMC pathogenesis. MiR-15 is reportedly associated with myocardial injury, inflammatory responses and viral infection. Whether miR-15 affects the occurrence and development of VMC remains largely unknown. The roles of miR-15 and their underlying mechanisms in CVB3-stimulated H9c2 cells were assessed in this study.

**Methods:**

We infected H9c2 cells with CVB3 to establish a VMC cellular model. We then determined the effects of miR-15 inhibition on three cardiomyocyte injury markers: lactate dehydrogenase (LDH), creatine kinase-MB (CK-MB) and cardiac troponin-I (cTn-I). The impact on CVB3-induced cell apoptosis and pro-inflammatory cytokines was also investigated. The effects of miR-15 inhibition on NLRP3 inflammasome activation were also assessed. The target relationship between miR-15 and NOD-like receptor X1 (NLRX1) was determined using a luciferase reporter assay.

**Results:**

MiR-15 expression was significantly upregulated in H9c2 cells after CVB3 infection. Inhibition of miR-15 significantly decreased the CVB3-induced levels of LDH, CK-MB and cTn-I. It also elevated cell viability, reduced CVB3-induced cell apoptosis and decreased the generation of the interleukins IL-1β, IL-6 and IL-18. Furthermore, we determined that miR-15 inhibition suppressed NLRP3 inflammasome activation by downregulating NLRP3 and caspase-1 p20 expression. We found a direct target relationship between miR-15 and NLRX1. Additionally, inhibition of NLRX1 reversed the protective effects of miR-15 inhibition against CVB3-induced myocardial cell injury by regulating the NLRP3 inflammasome.

**Conclusion:**

Our results indicate that miR-15 inhibition alleviates CVB3-induced myocardial inflammation and cell injury. This may be partially due to NLRX1-mediated NLRP3 inflammasome inactivation.

## Background

Viral myocarditis (VMC) can develop into dilated cardiomyopathy and heart failure. It is believed to be the principal cause of sudden cardiac death in children and young adults [1, 2]. Many viruses, including enteroviruses, adenoviruses and human herpes virus 6, are associated with VMC. Coxsackievirus group B type 3 (CVB3), which is an enterovirus of the picornaviridae family, is known as the main etiological agent in VMC [3, 4]. It can damage the myocardium and trigger excessive host immune responses, leading to myocardial injuries [5, 6].

Accumulating evidence indicates that immune response-mediated indirect injury is more deeply involved in the progression of VMC than direct virus-mediated injury [1, 7]. Several anti-inflammatory therapies, such as neutralizing the antibody against the interleukin IL-17 and blocking the MyD88 signaling pathway, can reduce inflammation and ameliorate the symptoms of VMC in mice [8, 9]. Despite these findings, the pathogenesis of VMC remains unclear, and no effective treatment method is available. It is still necessary to elucidate the mechanisms underlying the inflammation and immune reactions involved in VMC.

Innate immunity relies on pattern recognition receptors (PRRs) recognizing pathogen-associated molecular patterns (PAMPs), which are evolutionarily conserved [10]. PRRs include NOD-like receptors (NLRs), RIG-I-like receptors and toll-like receptors [11–13]. PRR-mediated innate immunity is deeply involved in the defense against viruses in cardiomyocytes [14].

However, aberrant innate immunity may cause disease [15]. NLRX1, as one member of the NLR family, is deeply involved in various diseases, including inflammatory diseases [16, 17], neurodegenerative disease [18] and cancers [19, 20]. However, its involvement and function in VMC is not well understood. It has been reported that NLRX1 negatively regulates the inflammation and innate immune response to viral infection [21, 22]. We speculated that NLRX1 may be an important checkpoint in the inflammation and injury of CVB3-induced VMC.

In recent years, microRNAs (miRNAs) have been identified as crucial for the regulation of gene expression at the transcriptional and post-transcriptional levels [23]. MiRNAs are 18–22 nucleotides in length and bind with the 3′-untranslated regions (UTRs) of mRNAs to induce either mRNA degradation or translation inhibition [24]. Dysregulation of miRNAs is part of the pathogenesis of various diseases, including VMC.

Dysregulation of miR-1, miR-21, miR-146, miR-155 and/or miR-221/− 222 is associated with VMC [25–28]. Moreover, CVB3 infection changes miRNA expression profiling in a mouse model of viral myocarditis [29, 30]. Using biological software, we predicted the miRNAs that directly target the 3′-UTR of NLRX1, focusing on miR-15 in this study. The expressions of members of themiR-15 family (including miR-16-1, miR-16-2, miR-195, miR-497, miR-15a and miR-15b) increase in many heart diseases [31, 32]. Hullinger et al. showed that inhibition of miR-15 alleviates cardiac ischemic injury [33]. MiR-15 is also reportedly associated with myocardial injury, inflammatory responses [33, 34], and viral infection [35]. However, little is known about the roles of miR-15 in CVB3-induced VMC.

We hypothesized that the miR-15–NLRX1 axis is involved in the development of VMC. We established a VMC cellular model by infecting H9c2 cells with CVB3. We verified the dysregulation of miR-15 and determined its effects on CVB3-induced injuries, including cell viability, apoptosis and inflammation. We also investigated the underlying molecular mechanisms of miR-15’s involvement in CVB3-induced myocardial cell injury.

## Materials and methods

### Cell culture

H9c2 cells, a clonal line originally derived from embryonic rat heart tissue, exhibit many characteristics similar to those of skeletal muscle myoblasts. They are often used in cardiomyocyte-related studies. H9c2 cells were obtained from the cell bank of the Chinese Academy of Sciences, originally coming from American Type Culture Collection (ATCC). They were cultured in Dulbecco’s modified Eagle’s medium (DMEM; Invitrogen Life Technologies) with 10% fetal bovine serum (FBS), 100 U/ml penicillin, and 100 μg/ml streptomycin (Invitrogen), and maintained in a 5% CO_2_ humidified atmosphere at 37 °C.

### CVB3 infection

CVB3 virus (Nancy strain) was purchased from ATCC. After HeLa cell-mediated amplification, the virus was titrated, and 100 TCID_50_ (TCID_50_ = 7.4, determined using the Reed-Muench method) was selected as the infective concentration. The H9c2 cells were randomly divided into the control and CVB3 groups. After washing 3 times with D-Hank’s that had been preheated at 37 °C, 0.6 ml of 100 TCID_50_ virus was added to the CVB3 group and the same volume of DMEM was added to the control group. For the CVB3 group, after culturing in an incubator for 2 h, the culture medium was removed and the cells were washed 3 times with D-Hank’s, and then 1 ml of DMEM containing 20% FBS was added to each well for further culture.

### Cell transfection

MiR-15 inhibitor, its negative control inhibitor (inhibitor-NC), miR-15 mimic and its negative control mimic (mimic-NC) were obtained from GenePharma. NLRX1 siRNA (si-NLRX1) and negative control siRNA (si-NC) were obtained from Sangon Biotech. Transfection with mimic, inhibitor or siRNA was done with Lipofectamine 2000 (Invitrogen). After 24 h of transfection, cells were treated with CVB3 for another 24 h.

### Quantitative real-time PCR

Total RNA was extracted from H9c2 cells using TRIzol reagent (Invitrogen). Reverse transcription was performed using a miRcute miRNA First-Strand cDNA Synthesis Kit (Tiangen Biochem). The quantitative real-time PCR was performed at least three times in triplicate using TaqMan microRNA assays (Applied Biosystems). The relative expression of miR-15 was analyzed using the 2^-ΔΔCt^ method by normalizing to U6 expression.

### Examination of myocardial markers

The levels of lactate dehydrogenase (LDH), creatine kinase-MB (CK-MB) and cardiac troponin-I (cTn-I) in the supernatants of cell lysates were measured using the available commercial kits (JianCheng Bioengineering Institute) with a fully automatic biochemical analyzer (Thermo Fisher Scientific).

### CCK-8 assay

Stably transfected and CVB3-treated cells were collected and at least three replicate experiments were performed in each group of cells. Generally, 10 ul CCK8 solution was added to each well at the indicated time points, and then incubated at 37 °C for 2 h. The absorbance was detected using a microplate reader at 450 nm. An increase in OD_450_ indicated increased cell viability.

### Flow cytometry

Cell apoptosis was assessed using an Annexin VFITC/PI Apoptosis Detection Kit (BD Biosciences). Briefly, after digestion with trypsin, the cells were resuspended in the binding buffer. After incubation with Annexin V-FITC and PI for 15 min without light, the apoptotic cells were identified using flow cytometry.

### Western blot

Total proteins were extracted from H9c2 cells after lysis in RIPA extraction buffer. Protein lysates were separated using SDS-PAGE, and then transferred onto PVDF membranes (Millipore). Then the membranes were blocked with 5% defatted milk for 1 h, followed by incubation with the following primary antibodies overnight at 4 °C: anti-Bcl-2, anti-caspase-3, anti-Bax, anti-NLRP3, anti-caspase-1 p20, anti-pro-caspase-1, anti-NLRX1 and anti-GAPDH. On the following day, after incubation with the corresponding secondary antibodies for 1 h at 37 °C, the membranes were visualized using the Odyssey Infrared Imaging System (LI-COR Biotechnology).

### Enzyme-linked immunosorbent assay (ELISA)

The levels of IL-1β, IL-6 and IL-18 in the cell culture supernatants were determined using ELISA kits (Beyotime Biotechnology) according to the manufacturers’ instructions.

### Luciferase reporter assay

MiR-15 inhibitor, inhibitor-NC, miR-15 mimic or mimic-NC were co-transfected with pGL3-NLRX1 3′-UTR (WT) or pGL3-NLRX1 3′-UTR mut (MUT) reporter plasmids that contained wild-type or mutant sequences within miR-15 binding sites, along with Renilla luciferase pRL-TK plasmids, into H9c2 cells. The luciferase activity was assessed using a Dual Luciferase Assay Kit (Promega).

### Assessment of caspase-1 activity

Caspase-1 activity was assessed using a caspase-1 assay kit (Beyotime Biotechnology). The absorbance was detected using a microplate reader at 405 nm.

### Statistical analysis

All experiments were performed at least three times in triplicate. All data were analyzed using the GraphPad Prism 5 software and results are shown as the mean ± SD. The comparisons of two groups were done with the *t* test, and the comparisons of multiple groups with one-way ANOVA followed by the Bonferroni test. *p* < 0.05 was considered statistically significant.

## Results

### Inhibition of miR-15 alleviated CVB3-induced myocardial cell injury

**Fig. 1:**
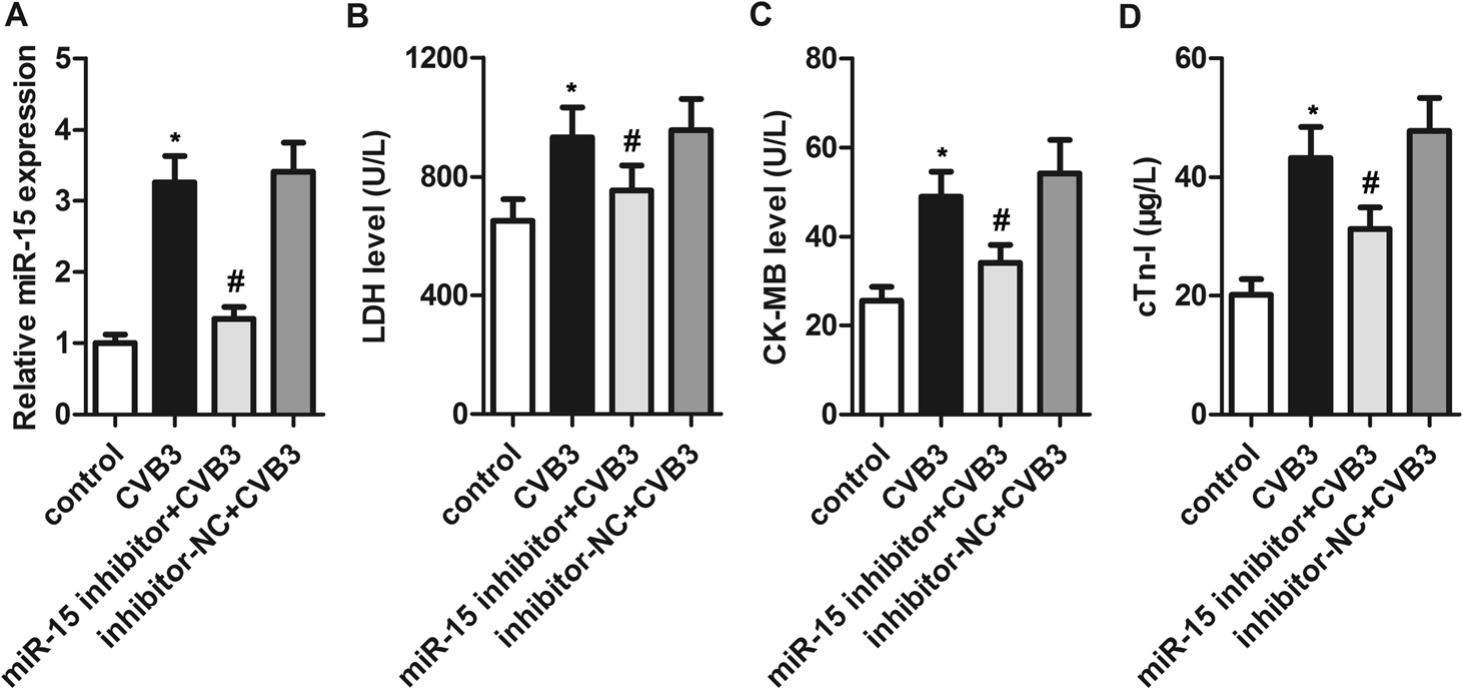
Inhibition of miR-15 alleviated myocardial cell injury induced by coxsackievirus B3 (CVB3). H9c2 cells were transfected with miR-15 inhibitor or inhibitor-NC for 24 h, and then infected with CVB3 for another 24 h. **a** – Expression of miR-15 was determined using quantitative real-time PCR and normalized to U6 expression. **b** through **d** – Lactate dehydrogenase (**b**), creatine kinase-MB (**c**), and cTn-I (**d**) levels in the supernatants of cell lysates were determined using a fully automatic biochemical analyzer. *p < 0.05 versus the control group, #*p* < 0.05 versus the CVB3 group

We infected H9c2 cells with CVB3 to establish a VMC cellular model and determined the expression of miR-15 in those cells using quantitative real-time PCR. CVB3 infection induced a significant increase in miR-15 expression compared with control cells (Fig. 1a), suggesting that miR-15 upregulation may have roles in CVB3-induced myocardial cell injury.

To explore the effects of miR-15, we transfected H9c2 cells with miR-15 inhibitor or inhibitor-NC and then infected them with CVB3. Transfection with miR-15 inhibitor significantly suppressed the CVB3-induced increase in miR-15 expression compared to the control.

To explore the effects of miR-15 on myocardial cell injury, we measured the levels of three cardiomyocyte injury markers: LDH, CK-MB and cTn-I. As expected, CVB3 infection markedly increased all three, implying that the virus induced injury. We found significantly lower LDH, CK-MB and cTn-I levels in the cells transfected with the miR-15 inhibitor prior to CVB3 infection (Fig. 1b through d). These results suggest that miR-15 inhibition could alleviate CVB3-induced myocardial cell injury.

### Inhibition of miR-15 promoted cell viability and suppressed CVB3-induced cell apoptosis

**Fig. 2:**
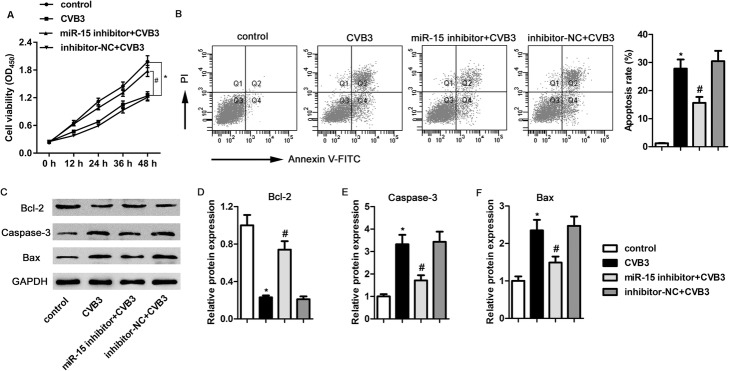
Inhibition of miR-15 promoted cell viability and suppressed cell apoptosis induced by CVB3. **a** – Cell viability was determined using the CCK-8 assay. **b** – Cell apoptosis was detected using flow cytometry. **c** through **f** – The protein expressions of Bcl-2, caspase-3 and Bax were determined using western blotting with corresponding histogram analyses, normalizing to GAPDH expression. *p < 0.05 versus the control group, #*p* < 0.05 versus the CVB3 group

We determined the effects of miR-15 on cell viability and apoptosis in CVB3-infected H9c2 cells. Compared with the control group, the cell viability in the CVB3 group markedly decreased and was elevated by the inhibition of miR-15 (Fig. 2a). We also assessed cell apoptosis using flow cytometry. Inhibition of miR-15 significantly reduced CVB3-induced apoptosis (by 27.82% in the CVB3 group and 15.61% in the miR-15 inhibitor+CVB3 group; Fig. 2b). The levels of apoptosis-related proteins were also of interest. As shown in Fig. 2c through f, the CVB3-induced decrease in Bcl-2 level was lessened after miR-15 inhibition. The increases in caspase-3 and Bax levels were significantly suppressed after miR-15 inhibition. These results suggest that miR-15 inhibition could promote cell viability and suppress CVB3-induced cell apoptosis.

### Inhibition of miR-15 reduced CVB3-induced inflammatory responses

**Fig. 3:**
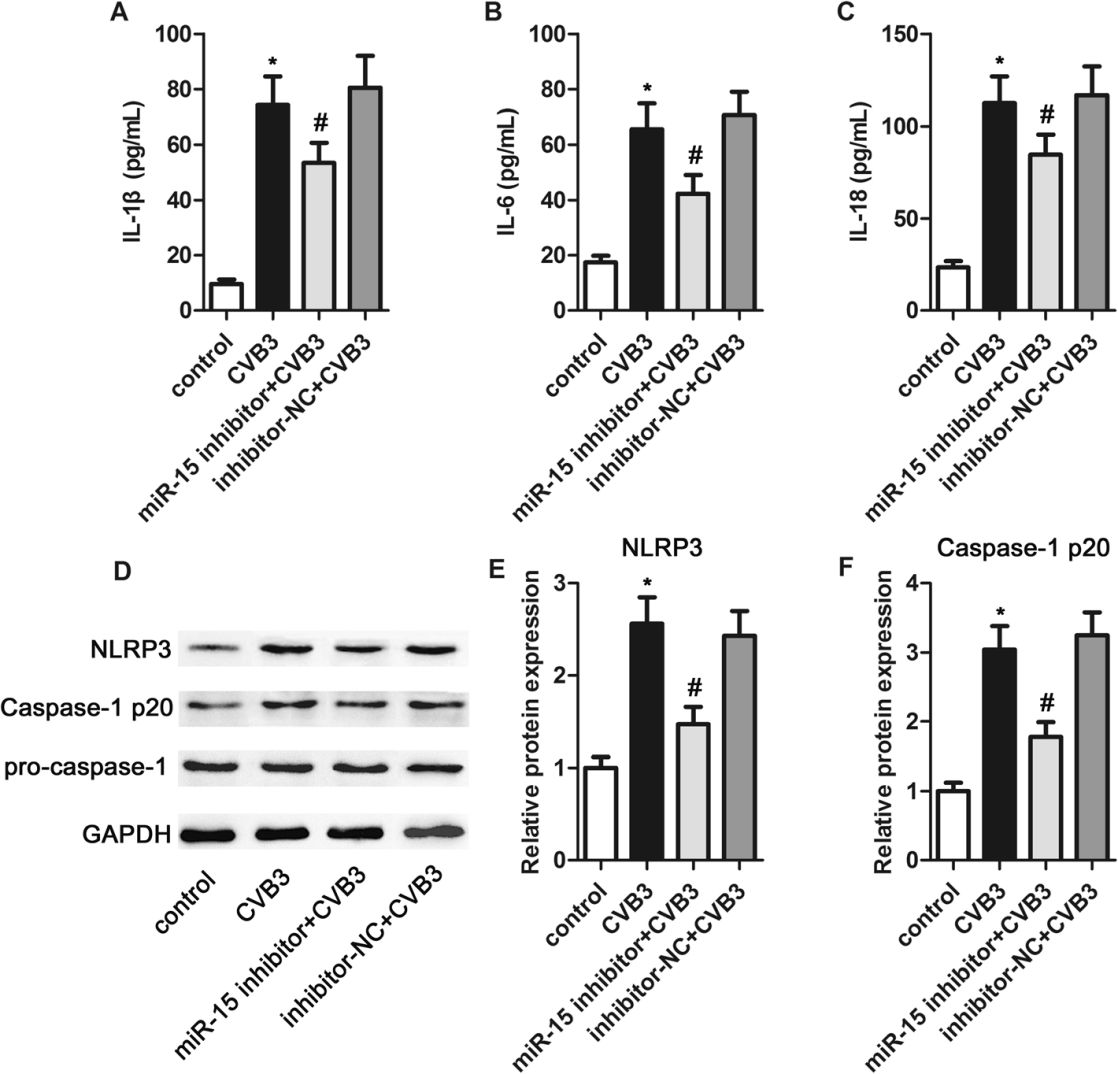
Inhibition of miR-15 reduced inflammatory responses induced by CVB3. **a** through **c** – The generation of IL-1β (**a**), IL-6 (**b**) and IL-18 (**c**) was determined using ELISA. **d** through **f** – The protein expressions of NLRP3, caspase-1 p20 and pro-caspase-1 were determined using western blotting with corresponding histogram analysis, normalizing to GAPDH expression. *p < 0.05 versus the control group, #*p* < 0.05 versus the CVB3 group

We next explored the effects of miR-15 on the generation of pro-inflammatory factors. Compared with the control group, the levels of IL-1β, IL-6 and IL-18 were markedly upregulated by CVB3 infection, while inhibition of miR-15 was found to suppress their production (Fig. 3a through c).

Previous studies have revealed that the production of pro-inflammatory factors is related to NLRP3 inflammasome activation, so we assessed the protein expression of NLRP3, pro-caspase-1 and caspase-1 p20. Compared with the control group, the levels of NLRP3 and caspase-1 p20 markedly increased in the CVB3 group, and these increases were suppressed by miR-15 inhibition (Fig. 3d through f). The level of pro-caspase-1 showed no obvious changes. These findings indicate that miR-15 inhibition could suppress CVB3-induced inflammatory responses, and that this may be attributed to the activation of the NLRP3 inflammasome.

### MiR-15 directly targeted NLRX1

**Fig. 4:**
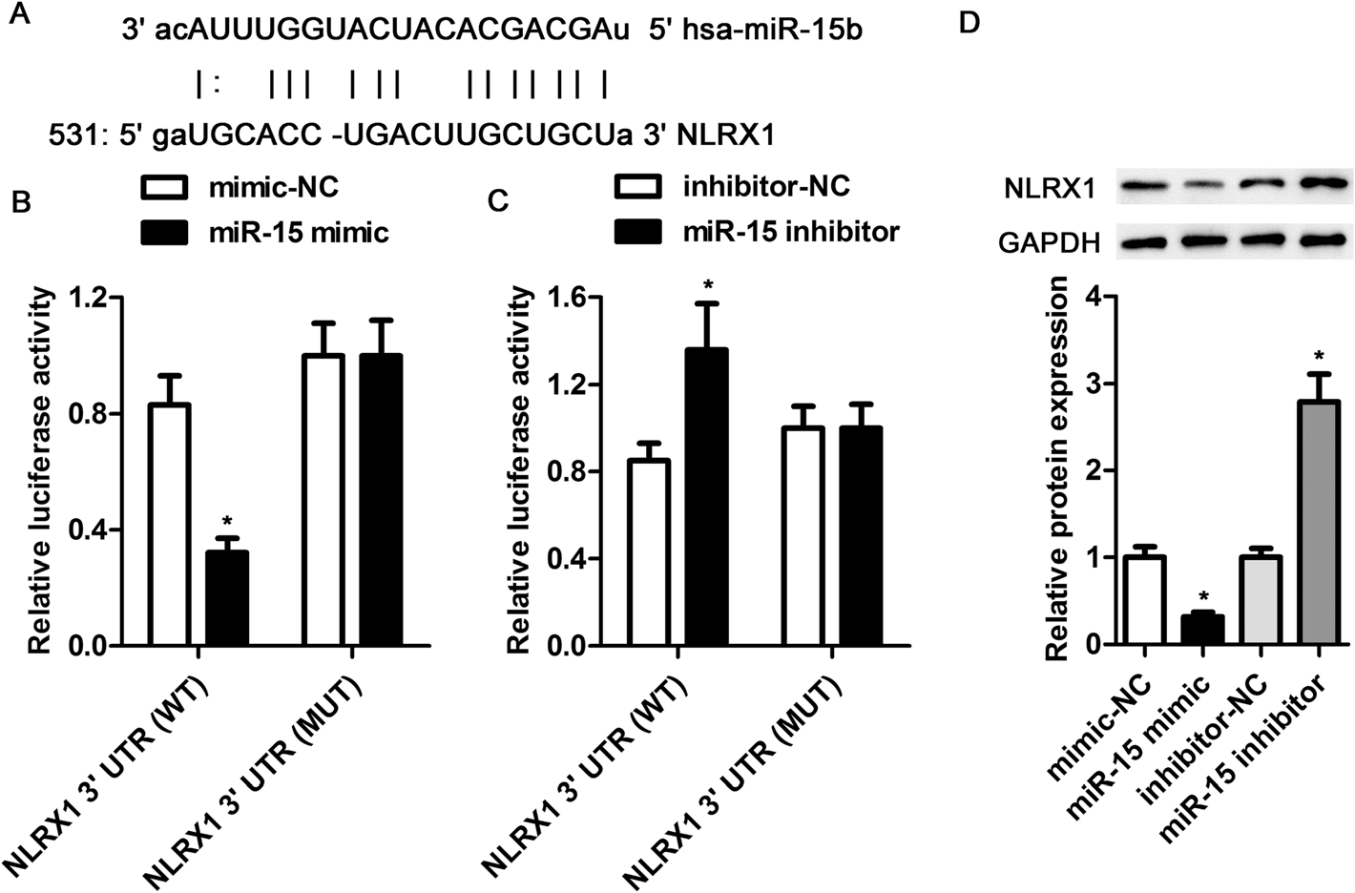
MiR-15 directly targeted NLRX1 to suppress its expression. **a** – The predicted miR-15 binding site in the 3′-UTR of NLRX1. **b** and **c** – MiR-15 mimic, mimic-NC, miR-15 inhibitor or inhibitor-NC were co-transfected into H9c2 cells with NLRX1 3′-UTR (WT) or NLRX1 3′-UTR mut (MUT) reporter plasmids, along with Renilla luciferase pRL-TK plasmids. Luciferase activity was analyzed 48 h post-transfection and normalized to the Renilla luciferase activity. **d** – The protein expression of NLRX1 in H9c2 cells stably transfected with mimic or inhibitor was determined using western blotting with corresponding histogram analysis, normalizing to GAPDH expression. *p < 0.05

The target relationship between miR-15 and NLRX1 was predicted using TargetScan and microRNA.org (Fig. 4a) and then confirmed using the luciferase reporter assay. Compared with the mimic-NC group, miR-15 overexpression markedly repressed the luciferase activity of the pGL3-NLRX1 3′-UTR (WT) plasmids that contained miR-15 binding sequences (Fig. 4b). However, miR-15 overexpression had no effect on the luciferase activity of pGL3-NLRX1 3′-UTR (MUT) plasmids that contained mutant miR-15 binding sequences. Compared with the inhibitor-NC group, miR-15 inhibition markedly elevated the luciferase activity of WT plasmids, but not that of MUT plasmids (Fig. 4c). A western blot assay found that miR-15 overexpression downregulated and miR-15 inhibition upregulated the protein level of NLRX1 (Fig. 4d). These findings indicate that miR-15 directly targets NLRX1 to suppress its expression.

### Inhibition of NLRX1 at least in part reversed the effects of miR-15 on CVB3-induced inflammatory responses

**Fig. 5:**
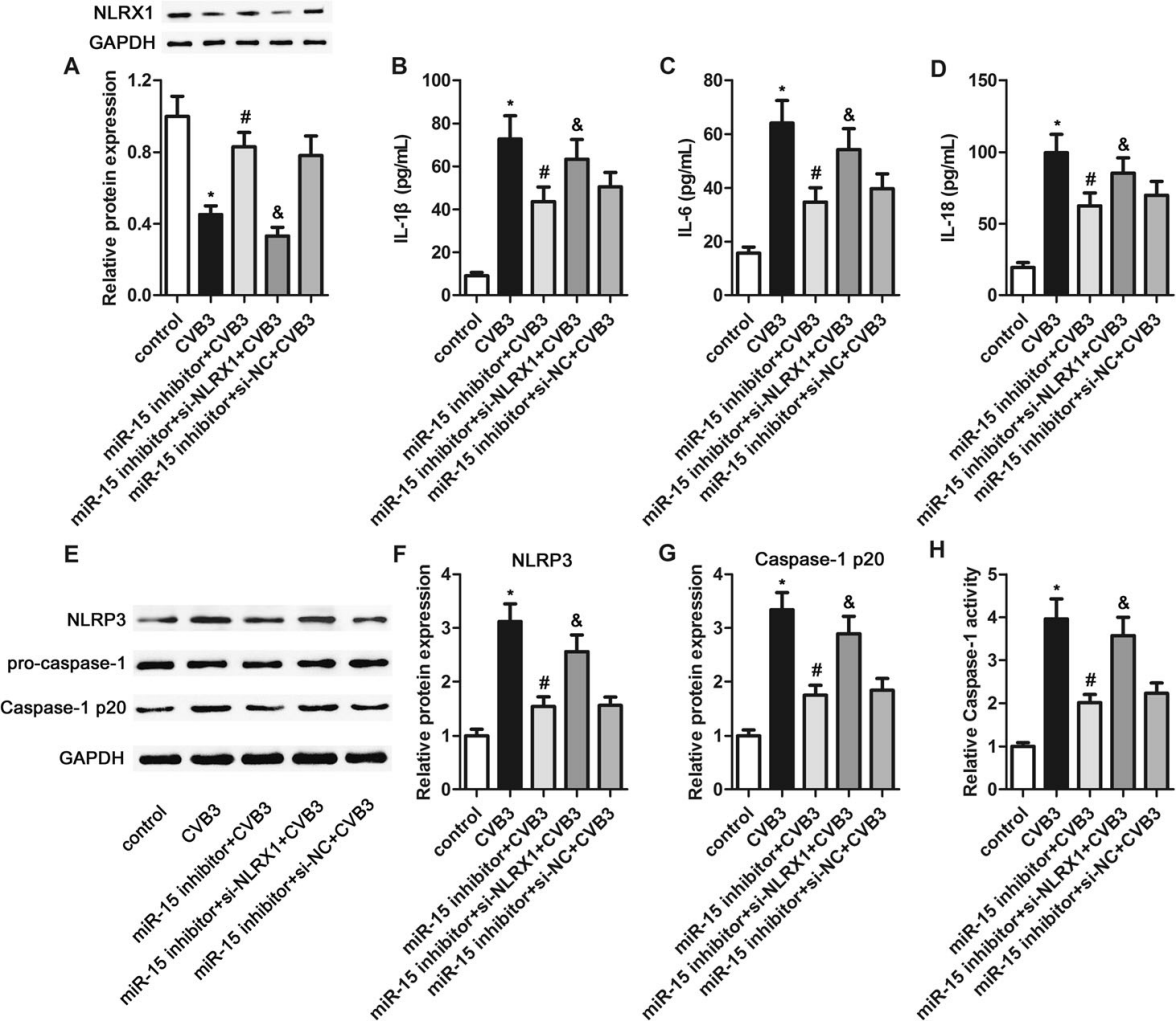
Inhibition of NLRX1 partly reversed the effects of miR-15 inhibition on NLRP3 inflammasome activation induced by CVB3. H9c2 cells were co-transfected with si-NLRX1 or si-NC with miR-15 inhibitor into H9c2 cells for 24 h, followed by CVB3 infection for another 24 h. **a** – The protein expression of NLRX1 was determined using western blotting with corresponding histogram analysis, normalizing to GAPDH expression. **b** through **d** – Production of IL-1β (**b**), IL-6 (**c**) and IL-18 (**d**) was measured using ELISA. **e** through **g** – The protein expressions of NLRP3, caspase-1 p20 and pro-caspase-1 were determined using western blotting with corresponding histogram analysis, normalizing to GAPDH expression. **h** – Caspase 1 activity was determined using a Caspase 1 Activity Assay Kit. *p < 0.05 versus the control group, #*p* < 0.05 versus the CVB3 group, and &p < 0.05 versus the miR-15 inhibitor+CVB3 group

To determine whether the effects of miR-15 in the CVB3-induced inflammatory responses are mediated by NLRX1, we co-transfected si-NLRX1 or si-NC with miR-15 inhibitor into H9c2 cells, followed by CVB3 infection. MiR-15 inhibition prevented the decrease in expression of NLRX1 induced by CVB3 infection (Fig. 5a), confirming that miR-15 directly regulates NLRX1. Compared with si-NC transfection, si-NLRX1 transfection significantly suppressed NLRX1 expression. We found that NLRX1 inhibition partly reversed the impact of miR-15 inhibition on the production of IL-1β, IL-6 and IL-18, with higher levels in the miR-15 inhibitor+si-NLRX1 + CVB3 group than in the miR-15 inhibitor+CVB3 group (Fig. 5b through d).

We next measured the expression of NLRP3 and caspase-1 p20, and found that their levels were markedly higher in the miR-15 inhibitor+si-NLRX1 + CVB3 group than in the miR-15 inhibitor+CVB3 group. This implies NLRP3 inflammasome activation by NLRX1 inhibition (Fig. 5e through g). Furthermore, caspase-1 activity was obviously higher after NLRX1 inhibition (Fig. 5h). These findings indicate that NLRX1 at least in part mediates the impact of miR-15 on CVB3-induced inflammatory responses and NLRP3 inflammasome activation.

### Effects of miR-15 in CVB3-induced myocardial cell injury can be partly attributed to NLRX1 inhibition

**Fig. 6:**
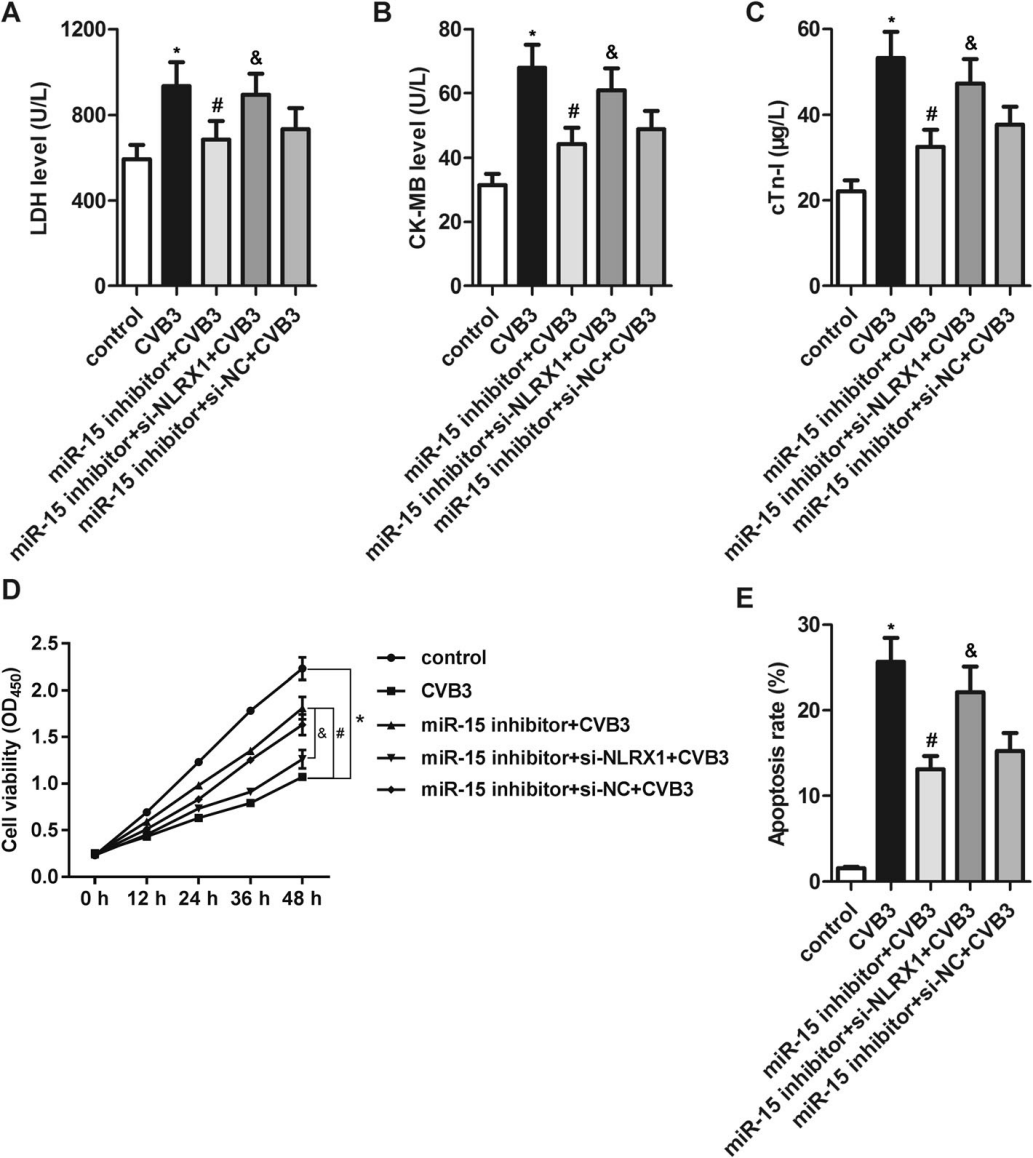
The effects of miR-15 in CVB3-induced myocardial cell injury can be partly attributed to NLRX1 inhibition. **a** through **c** – LDH (**a**), CK-MB (**b**) and cTn-I (**c**) levels in the supernatants of cell lysates were assessed with a fully automatic biochemical analyzer. **d** – Cell viability was determined using a CCK-8 assay. **e** – Cell apoptosis was detected via flow cytometry. *p < 0.05 versus the control group, #*p* < 0.05 versus the CVB3 group, and &*p* < 0.05 versus the miR-15 inhibitor+CVB3 group

Higher levels of LDH, CK-MB and cTn-I, decreased cell viability and increased cell apoptosis were found in the miR-15 inhibitor+si-NLRX1 + CVB3 group than in the miR-15 inhibitor+CVB3 group (Fig. 6), suggesting that NLRX1 at least partially contributes to the protective effects of miR-15 inhibition against CVB3-induced myocardial cell injury

.

## Discussion

CVB3 infection can cause significant injury to cardiomyocytes, leading to VMC, which is characterized by cardiac inflammation. It has been reported that virus-mediated direct injury and secondary immune reactions are both involved in VMC pathogenesis in animal and human models [36, 37], but the underlying molecular mechanisms are poorly understood. It has been reported that various miRNAs regulate VMC pathogenesis [29].

In this study, we assessed the roles of miR-15 in CVB3-induced myocardial cell injury and inflammation and investigated the underlying mechanisms. We found that CVB3 infection markedly upregulated miR-15 expression in H9c2 cells, indicating that upregulation of miR-15 may be involved in CVB3-induced myocardial cell injury. Previous research showed that CVB3 infection changed miRNA expression profiling in a mouse model of viral myocarditis [29, 30], but how CVB3 regulates miRNA expression is unclear. Some researchers proposed that this process is related to the activation of innate immune and antiviral pathways, such as the Toll-like receptor, NLR and JAK-STAT signaling pathways, and cytokine–cytokine receptor interaction [29]. However, its specific mechanism is still under investigation. The possible pathways or functional elements that may be involved in CBV3-induced upregulation of miR-15 will be explored in our future research.

We then used miR-15 inhibition to explore its roles in CVB3-induced H9c2 cells. First, we found that LDH, CK-MB and cTn-I markedly increased after CVB3 infection, implying CVB3-induced cardiomyocyte injury. Transfection with the miR-15 inhibitor lessened the increases in LDH, CK-MB and cTn-I induced by CVB3, indicating that miR-15 inhibition could alleviate CVB3-induced myocardial cell injury.

It has been reported that CVB3 infection induces apoptosis in HeLa cells by activating pro-apoptotic mediators [38]. Consistent with this published in vitro data, CVB3 infection in the heart activates cardiomyocyte apoptosis in both mice and humans [39–41]. Loss of cardiomyocytes due to viral infection may lead to cardiac disorder.

To explore the impact of miR-15 on CVB3-induced H9c2 cells, we assessed cell viability and apoptosis. We found that miR-15 inhibition significantly elevated cell viability, and reduced cell apoptosis. We also measured changes in apoptosis-related proteins. MiR-15 inhibition reversed the CVB3-induced decrease in Bcl-2 level and suppressed the increase in caspase-3 and Bax. These results suggest that the inhibiting miR-15 could promote cell viability and suppress CVB3-induced cell apoptosis.

Inflammation has been identified as the main reason for CVB3-induced myocarditis injury. Increasing evidence has shown that pro-inflammatory cytokines are critical in VMC [42]. Mice with VMC reportedly have increased pro-inflammatory cytokine levels, such as TNF-α and IL-1β [42, 43]. Other clinical studies have found elevated pro-inflammatory cytokines in patients with myocarditis [44, 45]. Among the cytokines, IL-1 signaling plays a crucial role in the induction of other pro-inflammatory cytokines, such as IL-6 and IL-18 [46, 47].

In this study, the production of IL-1β, IL-6 and IL-18 was significantly upregulated by CVB3 infection, which is consistent with previous findings. Also, miR-15 inhibition significantly suppressed the production of these cytokines, suggesting that inhibition of miR-15 could reduce production of pro-inflammatory cytokines.

We further investigated what molecular signaling mediated the anti-inflammatory effects of miR-15 inhibition. Inflammasomes serve as a platform for caspase-1 activation to modulate inflammatory responses [48]. Activated caspase-1 cleaves pro-IL-1 into a biologically active mature form and facilitates its release [49, 50]. Dysregulation of the NLRP3 inflammasome participates in various inflammatory diseases, including VMC [51–54]. In this study, CVB3 infection markedly increased NLRP3 and caspase-1 p20, indicating the activation of the NLRP3 inflammasome. These effects were suppressed by inhibition of miR-15. These findings indicate that miR-15 inhibition may suppress CVB3-induced inflammatory responses at least in part by inhibiting NLRP3 inflammasome activation.

We predicted that NLRX1 is one direct target of miR-15 and confirmed this using a luciferase reporter assay. We also found that miR-15 overexpression downregulated the protein level of NLRX1and miR-15 inhibition upregulated it. These findings indicate that miR-15 directly targets NLRX1 to suppress its expression. Considering the previously reported antiviral activity of NLRX1, we speculated that NLRX1 upregulation might mediate the protective effects of miR-15 inhibition against CVB3-induced myocardial cell injury. Moore et al. have indicated that NLRX1 may be a modulator, not a receptor, of pathogen-associated molecular pattern receptors [55]. NLRX1 also may negatively regulate RIG-I and toll-like receptors. NLRX1 is identified as a negative regulator of the NLRP3 inflammasome [56]. In this study, the suppression of NLRP3 inflammasome activation after miR-15 inhibition was partly reversed by co-transfection with si-NLRX1, suggesting that the effects of miR-15 on the NLRP3 inflammasome could be mediated by NLRX1. Furthermore, NLRX1 inhibition markedly suppressed the miR-15 inhibition-induced decrease in the production of IL-1β, IL-6 and IL-18. This finding confirms that the anti-inflammatory effects of miR-15 inhibition related to its regulation of the NLRP3 inflammasome were partly mediated by NLRX1. NLRX1 inhibition also was found to partly reverse the protective effects of miR-15 inhibition against CVB3-induced myocardial cell injury, characterized by increases in LDH, CK-MB and cTn-I, as well as decreased cell viability and increased cell apoptosis. These results indicate that the protective influence of miR-15 inhibition against CVB3-induced myocardial cell injury can be at least partly attributed to NLRX1-mediated NLRP3 inflammasome inactivation.

MiRNAs participate in a variety of biological processes through their wide range of target genes. Cimmino et al. showed that miR-15 and miR-16 act as natural antisense Bcl-2 interactors by negatively regulating Bcl-2 at a post-transcriptional level [57]. Thus, miR-15 and miR-16 may be important modulators of cell apoptosis. In this study, we showed that miR-15 played a crucial role in CVB3-infected H9c2 cells by targeting NLRX1. We speculate that the effect of miR-15 on VMC is due to the combined effects of the dysregulation of many target genes. The regulatory mechanisms underlying miR-15 in VMC need more investigation and the protective role of miR-15 inhibition in vivo should be further validated in future studies.

## Conclusion

These findings suggest that miR-15 dysregulation is closely associated with VMC and that miR-15 inhibition protects against CVB3-induced myocardial cell injury through modulation of the NLRX1-mediated NLRP3 inflammasome. Our study offers novel insights into the pathogenesis of VMC, indicating that the miR-15–NLRX1 axis may be a potential therapeutic target.

## Data Availability

The datasets used and/or analyzed during this study are available from the corresponding author on reasonable request.

## Abbreviations

CK-MB: creatine kinase-MB
cTn-I: cardiac troponin-I
CVB3: coxsackievirus B3
IL-18: interleukin-18
IL-1β: interleukin-1β
IL-6: interleukin-6
LDH: lactate dehydrogenase
miRNAs: microRNAs
NLRX1: NOD-like receptor X1
UTR: untranslated region
VMC: viral myocarditis
